# Need for Sexual, Reproductive, and Mental Health Promotion Among Diverse College Students in a COVID-19 Era

**DOI:** 10.1007/s10567-023-00460-5

**Published:** 2023-11-07

**Authors:** Michele Cooley-Strickland, Gail E. Wyatt, Tamra Burns Loeb, Lisa A. Nicholas, Amber Smith-Clapham, Amina Hamman, Misha Abraham, Enricka Norwood Scott, Graciela Albarran

**Affiliations:** 1 Semel Institute for Neuroscience and Human Behavior, Department of Psychiatry and Biobehavioral Sciences, David Geffen School of Medicine, University of California, Los Angeles, Los Angeles, CA 90024-1759 USA; 2 Department of Mental Health, Bloomberg School of Public Health, Johns Hopkins University, Baltimore, MD USA; 3 Department of Obstetrics and Gynecology, David Geffen School of Medicine at UCLA, Los Angeles, CA USA; 4 David Geffen School of Medicine, University of California, Los Angeles, Los Angeles, CA USA

**Keywords:** Sexual and reproductive health, Mental health, College students, COVID-19, Prevention, Sexual violence, Well-being, Diversity, Adolescents, Emerging adults

## Abstract

In 2020, the COVID-19 pandemic forced unprecedented disruptions in higher education operations. While the adverse mental health effects experienced by college students due to these changes are well documented, less is known about the impact on their sexual and reproductive health (SRH), and the reciprocal relationships between SRH and mental health among adolescents and emerging adults. This position paper reviews existing literature on the effects of the COVID-19 pandemic on SRH, sexual violence, unintended pregnancy, sexually transmitted illness and human immunodeficiency virus rates and highlights issues specific to college-aged males, females, racial/ethnic and sexual minorities, and individuals with disabilities. The need to conceptualize SRH as an integral component of normal development, overall health, and well-being in the context of COVID-19 is discussed. The need to prioritize the design and implementation of developmentally appropriate, evidence-based SRH interventions specifically targeting college students is identified. Furthermore, an intergenerational approach to SRH that includes parents/caregivers and/or college faculty and staff (e.g., coaches, trainers) could facilitate comprehensive SRH prevention programming that enhances sexual violence prevention training programs currently mandated by many colleges. Policies and programs designed to mitigate adverse pandemic-related exacerbations in negative SRH outcomes are urgently needed and should be included in mainstream clinical psychology, not only focused on preventing unwanted outcomes but also in promoting rewarding interpersonal relationships and overall well-being. Recommendations for clinical psychologists and mental health researchers are made.

## Introduction

In 2020, the COVID-19 pandemic forced unprecedented disruptions in higher education operations. In the U.S.A., over 1100 colleges closed their campuses and transitioned to online learning (Hess, 2020; Leistner et al., 2022). As a result, full-time college students were significantly more likely to live at home during the pandemic than pre-pandemic (Herbenick et al., 2022). While diverse negative mental health effects (i.e., depression, isolation, anxiety) experienced by college students due to these changes have been well documented (e.g., Browning et al., 2022; Lee et al., 2021), far less attention has been paid to the sexual and reproductive health (SRH) outcomes associated with the pandemic for this population (Leistner et al., 2022). These critical yet understudied aspects of health should be a focus of psychological research and clinical practice because, developmentally, adolescence and emerging adulthood are an important time for sexual exploration (Alexander et al., 2015). Increasing knowledge and skills that promote and protect health, save lives, and increase well-being in both the short and long term (Stoewen, 2017). More attention needs to be paid to the SRH of college students, particularly in the context of COVID-19 (Lindberg et al., 2020).

Many indicators of adolescent and college age health and well-being leading up to and into the pandemic—documented between 2011 and 2021—have worsened, including decreased protective sexual behaviors (e.g., condom use, sexually transmitted infection [STI] and human immunodeficiency virus [HIV] testing), mental illness, sexual violence, and suicidality (Centers for Disease Control and Prevention [CDC], 2023). Public health’s response to the pandemic necessitates attention to sexual health as a fundamental component of physical and mental health (Gleason et al., 2021). Renewed attention is needed by psychologists, parents, administrators, and health professionals in higher education to promote wellness, including the SRH of diverse college students. The adverse emotional and physical health outcomes of sexual and reproductive traumas caused by unintended pregnancy, contracting an STI or HIV, or experiencing sexual violence may be devastating for diverse youth and families, the impact of which may be worsened amid the COVID-19 pandemic.

This review aims to discuss the effects of the pandemic on SRH, sexual violence, unintended pregnancy, STI, and HIV rates, highlighting issues specific to college-aged males, females, racial/ethnic and sexual minorities, and individuals with disabilities. The purpose is to encourage a shift in psychology from a deficit model to a positive approach toward SRH as an integral component of normal development, overall health, and well-being for diverse adolescents and early adults. Recommendations are made to help mental health clinicians, educators, and researchers design and implement SRH prevention and health promotion interventions for college students in the COVID-19 era.

## College Relationships in the COVID-19 Pandemic Era

### Emerging Adulthood

American youth who are post-late adolescence and pre-early adulthood, aged 18–25, are classified as “emerging adults” (Arnett, 2000). About one-third of this population attend college and are semiautonomous in that they are still exploring their identities but do not yet feel like adults (Arnett, 2000; Goldscheider & Goldscheider, 1994). During this developmental stage, emerging adults have an increased capacity to regulate their emotions (Alexander et al., 2015; Arnett, 2000). There is a gradual transition to relationships that differ from those in earlier adolescence such that relationships last somewhat longer and are characterized by increased emotional and physical intimacy (Arnett, 2000). The reduction of parental involvement while living on a college campus enables students to spend sustained, unmonitored time with social and romantic partners, increasing opportunities to build intimacy.

### Campus Closures and Social Distancing

Attending college in the U.S.A. is a normative and long-anticipated positive experience for many youth. However, there is a need to understand and mitigate the cascading impact that the COVID-19 in-person campus closures and social distancing had on students’ socio-emotional, mental, and SRH (Son et al., 2020). For example, campus closures and social distancing restricted students:’ social opportunities; ability to meet new potential friends, sexual, and romantic partners; opportunities to deepen existing relationships; and accumulate experiences to help them prepare for the freedoms available on college campuses (Bowling et al., 2022; Leistner et al., 2022). College and health administrators focused on reducing the spread of COVID-19, yet little official guidance was provided about how to initiate and maintain intimate relationships while managing COVID-19 risks (Loeb et al., 2021). Only approximately 20% of college students began a new relationship during the pandemic (Chopik et al., 2023). Over half of college students reported decreases in sexual activity early in the pandemic, increased difficulties in “hooking up” (i.e., casual sexual encounters that do not carry expectation for future engagements; Maziarz & Askew, 2022), increased frequency of masturbation, and engagement in online dating (Leistner et al., 2022). Males were more likely than females to report that they increased frequency of consuming pornography, lacked intimacy, were unhappy with the frequency of sex they engaged in, and that the pandemic made it more difficult to find sexual partners (Maziarz & Askew, 2022).

Overall, college students reported decreases in quality of life, difficulties with obtaining basic resources, and increases in alcohol and cannabis consumption as a result of the pandemic (Firkey et al., 2022). This in turn may have made them vulnerable to sexual risk behaviors that threaten their health and well-being (CDC, 2016; Wong et al., 2019). These pandemic era changes in sexual and mental health patterns highlight the need for interventions that utilize contextually and developmentally informed strategies (Alexander et al., 2015). Currently, few evidence-based SRH interventions exist that are specifically targeted to college students (Alexander et al., 2015); even fewer integrate the effects of COVID-19.

### Pornography Consumption During COVID-19

Male and female college students consume pornography (e.g., 56.6% lifetime prevalence), primarily via internet technologies, with males reporting greater frequency than females (Camilleri et al., 2021). Students may also be interested in knowing that there is a positive association between excessive consumption of pornography—particularly that which is accompanied by masturbation—and the alarmingly high rate of erectile dysfunction (ED) in young men (Jacobs et al., 2021), termed Porn-Induced Erectile Dysfunction (Park et al., 2016). ED in young men has increased from rates between 2–5% in the late 1990’s and early 2000’s to 20–30% pre-pandemic (Nguyen et al., 2017). ED in middle-aged men is often attributed to physical causes, whereas ED in younger men is typically psychologically induced (e.g., relational problems, depression, anxiety, performance anxiety, addictive behavior; Awan et al., 2021; Papagiannopoulos et al., 2015).

Compulsive pornography use for male and female college students is significantly associated with “severe or very severe” depression (17.0%), anxiety (20.4%), and stress (13.5%; Camilleri et al., 2021), potentially reflecting unsatisfactory interpersonal relationships. Pornography consumption increases emerging adults’ sexual risk-taking behaviors, like casual hook ups, multiple sex partners, and extramarital sex (Braithwaite et al., 2015). Men who view pornography daily have nearly five times the number of lifetime sexual partners than non-viewers, a practice that is associated with sexual, reproductive, physical, and mental health risks (e.g., lack of condom use, smoking, alcohol and substance use, and poor physical and mental health; Braithwaite et al., 2010; Foster et al., 2012; Kuortti & Kosunen, 2009). A multinational survey of women showed that more frequent pornography use predicted greater sexual functioning during masturbation, yet had no adverse effects on sexual outcomes during partnered activity (McNabney et al., 2020). Predictors of orgasmic dysfunction for women were less education, anxiety, or depression (McNabney et al., 2020). COVID-19 pandemic pornography consumption increased from pre-pandemic rates (Awan et al., 2021; Maziarz & Askew, 2021). Braithwaite et al. (2015) reference the sexual scripts theory (Gagnon & Simon, 2005; Wright, 2011) and social learning theory in explaining how excessive pornography consumption may affect interpersonal relationships. It posits that sex is a biopsychosocial construct that is influenced by social norms, media, and personal experience, values, and attitudes dictating how one should proceed.

Research shows that that pornography promotes conceptualizing sex as instrumental (i.e., casual, uncommitted, recreational) and promotes hooking up. Now commonly ingrained in college student culture, hooking up reflects a shift from the previous model of dating and courtship to spontaneous, non-committed sexual engagements often with different partners heightening SRH risks (Bogle, 2008; Braithwaite et al., 2015). Braithwaite et al. (2015) conclude that because of the ease of access and frequency of pornography consumption among emerging adults, it cannot be viewed as merely harmless. Sexual scripts are being developed based on exposure to often misogynistic, patriarchal, and/or aggressive pornographic imagery. These sexual depictions may lack egalitarian relationships, perhaps forming beliefs and reinforcing behaviors that may contribute to unhealthy and dissatisfactory relationships, body images, and sexual practices. As adolescence is a highly vulnerable period when beliefs, attitudes, and behaviors about SRH are developed (Eryılmaz et al., 2021), more attention is warranted regarding the effects of pornography on youth and individuals with less sexual experience. SRH interventions may need to teach students about the subtle, oppressive influences of pornography (e.g., depiction of non-normative sexual organs prompting feelings of inferiority), as well as educate them about healthy, reciprocal, and rewarding interpersonal relationships as a counterbalance to popular—yet often unrealistic—pornographic scenarios.

## Reproductive Healthcare and Sex Education

The U.S. Supreme Court’s *Dobbs v. Jackson Women’s Health Organization* decision on June 24, 2022, resulted in cascading politicized restrictions in access to birth control, emergency contraception, and/or medical and surgical abortion in nearly half the states, heightening anxieties of vulnerable populations (e.g., youth, low income, racial/ethnic minorities, females) regarding essential components of reproductive health care (Stein et al., 2022). These uncertainties and restricted reproductive health choices have compounded an oppressive system; they add barriers that burden an already distressing health and social climate for teens and emerging adults as a result of the COVID-19 pandemic (Hertz et al., 2022; Jones et al., 2022) heightening the importance of comprehensive sex education.

### Sex Education and Youth

The mean age of first sexual intercourse is 17 years old (Martinez & Abma, 2020); 55% of 18-year-olds are sexually active (Abma & Martinez, 2017). Comprehensive sex education for youth has been found to positively affect contraceptive use (Cheedalla et al., 2020), reduce teen births, and lower risk for STIs (e.g., Kohler et al., 2008; Mark & Wu, 2022). Although 38 states (76%) and the District of Columbia mandate some form of sex and/or HIV education in public schools, only half of those require information on condoms or contraception and fewer require the information to be medically accurate (Guttmacher Institute, 2023). Despite declining rates in recent years (CDC, 2023; Martinez & Abma, 2020), the U.S.A. continues to have one of the highest teen pregnancy rates among affluent nations (Sedgh et al., 2015). Approximately half (49%) of all U.S. pregnancies are unintended and many will be terminated in abortion (Yee & Simon, 2010). In 2020, women in their twenties accounted for more than half (57.2%) of all abortions in the U.S.A. (Kortsmit et al., 2022). However, improving reproductive health literacy and access could decrease unintended pregnancies (Stein et al., 2022) and prevent resultant psychological and physical trauma.

### Sex Education and College Students

College campuses are highly populated areas of sexually and socially active young people (CDC, 2017; Lechner et al., 2013) who enter college with varying levels of health education (Vamos et al., 2018). Although academically exceptional, many students are poorly trained in caring for and managing their SRH (Lechner et al., 2013). This may be reinforced by the longstanding practice of using a deficit model in SRH prevention approaches on college campuses. Despite its prevalence, being sexually active still carries stigma and may reduce help-seeking behavior. Emerging adults and those younger may opt not to disclose SRH challenges to parents and other adults who may activate support. Parents vary in comfort and knowledge in being a resource to engage efforts to prevent unwanted outcomes (Walker, 2001); lacking education, some may worsen the effects of sexual secrets, especially in cases of sexual violence.

Often having received abstinence-only education or no formal sex education in high school—perhaps in part an attempt to discourage discussions of the effects of vaginal penetration, ovulation, and insemination and/or sexual identity and orientation (Wyatt, 1992)—college students frequently enter college with inaccurate or insufficient sexual health information (Bailey, 2016; Cassidy et al., 2018). Compared with upper-class students, first-year undergraduate students are less likely to access campus sexual health services, know they exist, or reasons to use campus SRH centers (Cassidy et al., 2018). Non-heterosexual students are less likely to access sexual health services and understand their sexual risks than heterosexual students, highlighting the need to target this vulnerable population (Cassidy et al., 2018). LGBTQ+ centers are helpful in this regard, but diverse college communities would benefit from a more integrative approach where sexual minority students felt welcomed, understood, and empowered in SRH promotion campus programming. The same is true for racial/ethnic minority students and students with disabilities, all of whom have disproportionate rates of STIs, HIV, and sexual violence and historically been oppressed and/or experienced disparities in health promotion services. Promoting community solidarity, accurate SRH information and skills may minimize risks and improve diverse students’ sexual health (Bailey, 2016).

## HIV/STI Rates in College Age Students

Approximately, half of new sexually transmitted infections (STIs) are among high school and college age people (aged 15–24 years; CDC, 2022a). Adolescents and emerging adults have high rates of STIs and low rates of condom use, increasing their chances of contracting or transmitting HIV (Petsis et al., 2020). STI and HIV testing among this age group is low, despite 15- to 24-year-olds accounting for disproportionate rates of syphilis, gonorrhea, and chlamydia (i.e., 22%, 42%, and 62%, respectively, compared to representing only 13% of the population; CDC, 2019; Trepka & Kim, 2010). STI rates for college students are higher than same-aged peers not enrolled in college and may be higher among students attending four-year colleges than those attending two-year colleges (Habel et al., 2016). Although, only 20.2% of female college students were tested for STIs in a one-year period, the testing rate for females was two and one-half times higher than male college students (7.7%; Cuffe et al., 2016). As such, STI and HIV rates may be underestimated in college populations. Research with college students has shown that they lack accurate information about the risk for STIs, transmission, and prevalence (Downing-Matibag & Geisinger, 2009). For example, only 30% knew that asymptomatic individuals may be positively diagnosed and nearly two-thirds (64%) were unaware of names of STIs beyond HIV (Subbarao & Akhilesh, 2017).

In 2019, adolescents and emerging adults—who accounted for 21% of the 36,801 diagnoses of HIV infection—were least likely of any age group to be aware of their HIV infection status, stay in care, or have a suppressed viral load (CDC, 2022c, 2022d). Recent infection and low testing rates may contribute to a lack of awareness of HIV status (CDC, 2023b). Persons who are unaware they have STIs or HIV do not get medical care or receive treatment and can unknowingly infect others while increasing their likelihood of more severe health outcomes (e.g., infertility, cancer, poor pregnancy, and birth outcomes; CDC, 2022c; World Health Organization, 2019). It is important to help college students understand their risk for infection and practical ways to prevent exposure and transmission.

### HIV/STI Rates in Special Populations

#### Ethnic/Racial Minorities

From a historical context, significant racial/ethnic differences in STI and HIV rates are associated with health disparities, including bias, racism, and decreased reproductive health resource access (Sutton et al., 2021). Ethnic and racial minority youth enrolled in colleges are at high risk for unwanted sexual health outcomes (LeBlanc et al., 2014). For example, African American and Hispanic American men were less likely to be familiar with most methods of contraception compared to men from other ethnic backgrounds (Borrero et al., 2013). Even after controlling for socioeconomic factors (e.g., education, income, access to health care), racial and ethnic disparities persist in knowledge of contraceptives (Rosenfeld et al., 2017), rates of unintended pregnancies (Gaydos et al., 2010; Grady et al., 2015), STI screening (Pratte et al., 2018; Tangka et al., 2017), and mental health care (Cook et al., 2017). African American and Hispanic American adolescents and emerging adults have the highest rates of HIV transmission in the U.S.A. (CDC, 2022a), consistent with the higher prevalence of STIs and HIV within their communities. For example, although African Americans represent only 12% of the U.S. population, 32% of all cases of chlamydia, gonorrhea, and syphilis were diagnosed among African Americans, and they account for 42.1% of all new HIV diagnoses (CDC, 2020). Hispanic Americans represent 19% of the U.S. population yet comprise 27% of new HIV diagnoses (CDC, 2020). Hispanic and African American cases combined make up more than half of reported HIV cases in the U.S.A. (*HIV & AIDS Trends and U.S. Statistics Overview,* 2022). Among American females living with diagnosed HIV infection in 2019, 57% were African American, 21% Hispanic American, 16% European American, 5% multi-racial, 1% Asian American, and 1% or less were American Indian/Alaska Native and Native Hawaiian/Other Pacific Islander (CDC, 2022a). HIV infection was attributed to heterosexual contact in 93% of Asian American females, 91% of African American females, and 87% of Hispanic American females (CDC, 2022a). From a historical context, significant racial/ethnic differences in STI and HIV rates are caused by structural barriers and disparities, including bias, racism, discrimination, poverty, and decreased access to high-quality reproductive health resources needed for prevention or treatment (CDC, 2019; Kates et al., 2018; Sutton et al., 2021).

#### Sexual Minorities

Estimates range widely in the rates of American adults who self-identify as lesbian, gay, bisexual, transgender, and questioning (LGBTQ+; i.e., 2.8–4.1%) and are substantially higher (i.e., 10%) when including individuals who have participated in same-sex behavior but do not identify themselves as lesbian, gay, or bisexual (Kates et al., 2018). Including individuals who participate in same-sex behavior without identifying as LGBTQ+ is particularly relevant during the exploratory phase of adolescence and emerging adulthood. PEP regarding STI and HIV rates among LGBTQ+ individuals, the highest percentage of HIV infections were attributed to contact between men who have sex with men (MSM; 66% overall and 81% among males; HIV & AIDS Trends and U.S. Statistics Overview, 2022). MSM are at elevated risk for gonorrhea, syphilis, and hepatitis (Kates et al., 2018). Of the over one million Americans who identify as transgender, 2% comprise new HIV cases (HIV & AIDS Trends and U.S. Statistics Overview, 2022). New infection rates declined 8% from 2015 to 2019, largely attributable to substantial reduction in HIV rates among young gay, bisexual, and other MSM (HIV & AIDS Trends and U.S. Statistics Overview, 2022). During this period, new infections among adolescent and emerging adult MSM were reduced by one-third (33%; HIV & AIDS Trends and U.S. Statistics Overview, 2022) in part due to advances in HIV prevention (e.g., Pre-Exposure Prophylaxis [PrEP]; Post-Exposure Prophylaxis [PEP]).

PrEP is a daily oral pill or bimonthly injection (Apretude) containing antiretroviral drugs that are highly effective in preventing the acquisition of HIV infection (Huang et al., 2018). Declines in HIV were observed in young men of all racial/ethnic groups; however, African American and Hispanic American MSM remain disproportionately affected with significantly elevated rates (HIV & AIDS Trends and U.S. Statistics Overview, 2022) in part because preventive efforts have been targeted primarily toward European American MSM. For example, despite indicators recommending PrEP for African Americans in one study accounting for almost 40% of the sample, nearly six times as many European Americans than African American people were prescribed PrEP (Smith et al., 2018). More equity and empowerment in the allocation of HIV preventive resources is essential, particularly that which is tailored as developmentally and culturally relevant (e.g., accounts for historical medical mistrust, removes structural and perceived barriers for women and racial/ethnic minorities; Chandler et al., 2022). Female-to-female sexual transmission of HIV is extremely rare, with only one reported U.S. case (CDC, 2014). Nonetheless, lesbian and bisexual women remain at risk for HIV and STIs (Kates et al., 2018). There are significant racial, ethnic, and gender gaps in effective STI and HIV preventive implementation efforts (Huang et al., 2018) that need to be remedied. A potentially important risk factor in HIV transmission is sexual violence. There is evidence for a significant and reciprocal relationship between sexual violence and HIV transmission risk (Klot et al., 2013).

### Barriers to HIV/STI and COVID-19 Health Services Utilization

Research conducted pre-pandemic showed a strong association between students’ social opportunities and motivations for seeking sexual health services (Cassidy et al., 2018). However, students reported numerous barriers to sexual health service utilization, including lack of or limited knowledge about sexual health and its relationship to overall health (e.g., unsure of why they would need sexual health services), as well as access, resources, referrals, stigma (Cassidy et al., 2018; Leistner et al., 2022), health insurance coverage, cost, and transportation (Cassidy et al., 2018; Moilanen et al., 2010). In response, flexible hours of operation, convenient locations, and mobile clinics to increase access were encouraged (Cassidy et al., 2018). However, the pandemic interfered and in some cases, stopped access; it adversely affected trends in STIs, reflecting a strained public health infrastructure (CDC, 2020). COVID-19-driven college campus closures severely limited access to campus-based SRH services and restricted opportunities for students to increase sexual health knowledge and learn about risk prevention. Pre-existing barriers were likely to have been exacerbated by students’ absence from campus, resource reallocation (e.g., COVID-19 testing and treatment), and shift to remote clinic operations, necessitating a new need to identify, implement, and evaluate sexual and reproductive preventive health programming and resources for college students since the pandemic onset.

## Sexual Violence on College Campuses

Pre-COVID-19 pandemic, the Association of American Universities reported that 13% of all undergraduate and graduate students experienced some form of sexual violence (i.e., nonconsensual sexual contact by physical force or inability to consent) while enrolled in college, whereas nearly 25% of female college students were victims of sexual assault or misconduct (Cantor et al., 2020). The report compared data from 2015 to 2019 and found an increase in the occurrence of acts of sexual violence on college campuses highlighting the importance of sexual violence prevention programs for college students. Adolescent sexual violence rates continued to increase from pre-pandemic to the COVID-19 lockdown/stay-at-home mandates (i.e., 2019–2021), albeit modestly at 1.5% (CDC, 2023). The modest increase may because adolescents likely resided in their familial home with restricted opportunities for romantic and sexual relationships. Research has shown that female students are more likely to experience rape or sexual assault victimization when they are away from home (Sinozich & Langton, 2014). During the pandemic, college students may have lived at home with their families, on campus, or stayed with roommates or domestic partners off campus thereby affecting their susceptibility to sexual violence—perhaps inversely proportionate to their amount of parental supervision and monitoring.

College students are at an increased risk of sexual violence during the first few months of their first and second semesters in college (Kimble et al., 2010) when they are freshly away from home and least familiar with campus social culture and relevant contexts to be able to effectively protect themselves (Amar et al., 2014). Significant numbers of college students experience some form of sexual violence during their time on campus (Fedina et al., 2016), with college students’ rates of sexual assault being higher than the same-aged national average (Garcia et al., 2012). For example, male college students (aged 18–24) are 78% more likely than non-students to experience rape or sexual assault (Sinozich & Langton, 2014). It is estimated that one in five (20%) women on college campuses has been victimized, with women aged 18–24 at elevated risk of sexual violence (Humphreys & Towl, 2020; Sinozich & Langton, 2014). In most cases, weapons were rarely (10%) involved, but the offender was known to the victim in about 80% of rapes and sexual assaults on campus (Sinozich & Langton, 2014). Females, underclassmen, racial/ethnic minorities, sorority women, students with disabilities, and students with past histories of sexual victimization were found to have higher prevalence of sexual violence rates than men and upper-class students (Cantor et al., 2020; Fedina et al., 2016; Holloway et al., 2022; Krebs et al., 2007). International students are also at elevated risk for sexual violence (Holloway et al., 2022), particularly given the cultural and language differences they encounter while attending college in a foreign country.

### Sexual Violence Rates in Special Populations

#### Ethnic/Racial Minorities

The CDC National Intimate Partner and Sexual Violence 2010 summary reports that approximately 34% of multi-racial women, 27% of Alaska Natives/American Indian women, 22% of African American women, 16% European American women, and 14.6% of non-White Hispanic American women report having experienced some form of sexual violence. Limited data are available to determine accurate rates of sexual violence within Asian American and Pacific Islander (API) communities (Tjaden & Thoennes, 1998), although estimates project 33% of API women have experienced sexual assault (Tsong & Ullman, 2018). Elevated rates of sexual violence may occur in marginalized racial/ethnic populations for many reasons, including the influence of historic socialization. For example, sexual assaults of African American men, women, and children were legal, profitable, and systematized during slavery; many remained silent about this cruelty and abuse in order to survive (Wyatt et al., 2023). Influences that are more contemporary may include normalization to conform to the will of partners with toxic aggression, *machismo*, or masculinity. In less affluent communities, there may be less privacy, increased surveillance by law enforcement, or increased involvement of social services resulting in more oversight by mandated reporters and higher recorded rates of sexual violence (Smith-Clapham et al., 2023).

#### Sexual Minorities

Although the vast majority of research on college sexual violence involves heterosexual students (Ray et al., 2021), sexual minority college students are at a higher risk of experiencing sexual violence when compared to their cisgender heterosexual peers (Martin-Storey et al., 2022). Compared with heterosexual males and females and sexual minority males, sexual minority females have the highest rates of college student sexual victimization (58%; Ray et al., 2021). Prevalence rates for sexual assault were three times higher for male sexual minority college students than heterosexual males (Coulter et al., 2017). In comparing sexual violence victimization rates between LGBTQ+ and heterosexual individuals, 13.2% of bisexual men and 11.6% of gay men reported being a victim of sexual violence compared to 1.6% of heterosexual men (Balsam et al., 2005). Nearly half (46%) of bisexual women compared to 13% of lesbians and 17% of heterosexual women reported having experienced sexual violence (Walters et al., 2013). These discrepancies in sexual assault rates were slightly lower among college students (i.e., bisexual female college students experienced rates twice as high as heterosexual women; Coulter et al., 2017). Over one-fourth of transgender individuals reported being sexually assaulted after the age of thirteen (Testa et al., 2017).

A recent study of college students found that risk factors for sexual assault victimization include heavy alcohol consumption and a history of child sexual abuse for both heterosexual and sexual minority students, whereas sexual risk-taking behaviors (e.g., having multiple sexual partners) was only a risk factor for heterosexual—but not sexual minority—students (Ray et al., 2021). Involved in nearly half of all cases, alcohol use—whether by the perpetrator, survivor, or both—is a significant risk factor for sexual assault (Abbey, 2002). This may be in part because the victim is less prepared to ward off potential offenders and intoxicated perpetrators are less inhibited in their behaviors (McGraw et al., 2022). Hooking up is also a risk factor for college student sexual victimization (Sutton et al., 2021). Regardless of individual lifestyle choices made by the victim, the perpetrator bears the sole responsibility for sexual violence (McGraw et al., 2022). The intersection of gender and sexual orientation is important to focus on in sexual violence research and practice given that the combination yields unique stressors (Meyer, 2015) and lifestyle choices; focusing on the gender dichotomy (i.e., male vs. female) is inadequate (Ray et al., 2021). Sexual minority college students represent a vulnerable population for whom research and preventive interventions need to explicitly focus, including LGBTQ+-specific sex and relationship education, increased access to SRH and mental health resources, and strategies to reduce risk factors and enhance protective factors for STIs, HIV, and violence prevention among sexual minority individuals.

#### College Students with Disabilities

Federal law prohibits college admissions departments from discriminating against applicants by numerous factors, including disabilities. As such, college students are a diverse population and include emerging adults with various forms of disabilities. Historically, people with disabilities were mistreated in reference to their reproductive health. For example, forced sterilization remained legal in many states into the 1970s (Holmes, 2021). The Individuals with Disabilities Education Act in 1975 led to the development of the first formal sex education curriculum for people with disabilities. Students with disabilities are entitled to receive all programs and services available to other students, and they perhaps have greater needs given that people with disabilities have an increased risk of experiencing sexual violence compared with those without disabilities (CDC, 2022b).

Sexual violence (e.g., rape, stalking, intimate partner’s control of sexual, or reproductive health) is more likely to occur in females with a disability than those without (CDC, 2022a). A study on the rates of crimes against individuals with disabilities suggested that 18- to 24-year-olds were at high risk of being a victim of sexual violence (Harrell, 2012). Avoiding educating people with disabilities on their SRH is largely due to the assumption that they are asexual or have limited interest in sexual activity (Long-Bellil, 2022). Lack of access to comprehensive sexual education can make college students with disabilities even more vulnerable to sexual abuse, STIs, and unintended pregnancy (Long-Bellil, 2022). Colleges need to remove barriers that affect people with disabilities, including using inclusive approaches to improve the health, well-being, and engagement of people with disabilities in college life (CDC, 2022b).

### Barriers to Reporting Sexual Violence

The social dynamics of college students on campus fosters environments that increase susceptibility rates for vulnerable groups when it comes to sexual violence. Incapacitated assault is defined as sexual assault where the survivor is sexually abused while under the influence of alcohol or drugs, passed out, or otherwise incapacitated (Kilpatrick et al., 2007). Campus context matters, as 58% of incapacitated rapes and 28% of forced rapes took place at a party (Krebs et al., 2007). Awareness of what constitutes sexual assault is an issue, as nearly three-fourths (70%) of college students did not realize they were being abused until after the incident occurred (Libertin, 2017; Network for Victim Recovery of DC, 2020). It may be years later when they recognize they were the victims of sexual assault, although the effects of the trauma may have had an immediate onset (Wyatt, 1992).

Under-reporting to authorities is a serious problem on college campuses, with only 20% of college student rape and sexual assault survivors reporting the assaults to police, a rate that is lower than same-aged non-student peers (32%; Sinozich & Langton, 2014). The primary reasons for reporting include to protect others from the offender and prevent recurrences or escalation of violence (Rape, Abuse & Incest National Network [RAINN], 2023). Sexual violence may elicit an emergency trauma response, such as Fight, Flight (run away), Freeze (stay), and Fawn (appease) (Steinman, 2022). The students who did not report their abuse could be classified as having chosen “freeze” or “flight”; they may not have known the available options on campus for making a report and/or may have feared their attacker would retaliate. The majority chose “fawn,” pretending as if there were no problem so their attacker would desist or fail to acknowledge or recognize that there was a significant problem (Network for Victim Recovery of DC, 2020).

Systemic issues affect whether college students officially report sexual violence experiences to authorities. For example, few (9%; RAINN, 2023) *reported* cases result in felony conviction of the perpetrator thus reducing confidence in the effectiveness of the system and increasing fear of retaliation. Media and social media have been forums where survivors have been publicly ridiculed and persecuted, thus creating a culture of shame that college student survivors may try to avoid. Some may choose to report to a confidential resource like their campus Title IX office or residential life rather than campus or municipal police. Historical mistrust of law enforcement and judicial systems is another reason they may not report to authorities, particularly if they are a person of color (Smith-Clapham et al., 2023). Fear and apprehension of reporting these violent incidents complicates the ability to address and care for students who have been traumatized (Carey et al., 2018). Plentiful resources, clear communication mechanisms, and easy access are critical elements in addressing these problems frequently occurring in college campus life (Halstead et al., 2017).

## Sexual Violence: Mental and Physical Health Outcomes

The COVID-19 pandemic exacted a tremendous burden on adolescents who already suffered from depression, anxiety, and thoughts of suicide before it began (St. George, February 13, 2023). Depression, stress, and anxiety are risk factors for sexual violence, as are alcohol, drug use (Libertin, 2017), and engaging in casual or frequent hook-ups (Sutton & Simons, 2015). Alternative means of coping and managing the trauma may include substance abuse, social isolation, and emotional withdrawal (Griffin et al., 2022; Kaukinen, 2014). College students with histories of trauma, violence, and perceived discrimination are at greater risk of sexual violence (Bhochhibhoya et al., 2021; Hébert et al., 2019; Kaukinen, 2014). Adolescent sexual violence increased from the pre-pandemic to the pandemic period (i.e., 2019–2021; CDC, 2023).

### Sexual Violence Physical Health Outcomes

Trauma occurring from sexual or dating violence may have long-lasting and detrimental consequences, especially on emerging adults whose prefrontal cortex are still developing (Carey et al., 2018; RAINN, 2023). College student survivors report higher rates of mental health issues related to an attack than non-students (Kilpatrick et al., 2007). Adolescents and emerging adults are vulnerable to the known psychosocial complications of engaging in nonconsensual sexual encounters and the future trauma that manifest in chronic health conditions. The more commonly referenced complications are unintended pregnancy, STIs, HIV, and abortion (Wyatt, 1992); however, chronic gastrointestinal disorders (e.g., irritable bowel syndrome) may also occur in women who report a history of sexual assault (Drossman et al., 1990; Pemberton & Loeb, 2020). Survivors may experience chronic pelvic pain (Rapkin et al., 1990), infertility, sexual dysfunction (Reed et al., 2000), and pelvic floor disorders (Cichowski et al., 2013) that negatively impact quality of life years beyond the initial assault. These resultant conditions may cause serious economic consequences, including financial stress directly on the student survivors, as well as on college and public health care systems already burdened by the intractability of COVID-19 to provide the appropriate mental and medical services (VOGT et al., 2022).

### Sexual Violence: Mental Health Outcomes

Turning to alternative means of coping and managing the trauma may involve social isolation and emotional withdrawal (Griffin et al., 2022). Severe mental health outcomes may include post-traumatic stress disorder (PTSD), eating disorders, suicidal ideation, anxiety, depression, distress, substance, and alcohol abuse (Carey et al., 2018; Chang et al., 2015; Dilip & Bates, 2021; Oh et al., 2021). LGBTQ+ students, especially trans students, are at highest risk for sexual assaults and severe mental health outcomes associated with the attacks (Kammer-Kerwick et al., 2019; RAINN, 2023). Depressive symptoms increased in college students who experienced sexual assaults; they also experienced dysfunction in basic psychological needs, such as competence, autonomy, and relatedness (Chang et al., 2015). Regarding academic performance, students who suffered from a sexual assault reported PTSD symptoms including difficulty focusing, concentrating, and retaining information potentially leading to lower academic grades and an increase in dropout rates prior to graduation (Auerbach et al., 2016; Molstad et al., 2023). Survivors often blame themselves, few disclose the abuse out of fear of shame or stigma, and many do not seek mental health services resulting in an exacerbation of mental health problems (Dilip & Bates, 2021; Libertin, 2017).

Students who experience sexual trauma, loneliness, and anger are more likely to develop suicidal ideation and engage in self-harming behaviors (Keefe et al., 2018; Par, 2020). Suicide is rated the second highest cause of death for college students, with sexual assaults among the primary reasons for suicidality (Oh et al., 2021). Over 30% of women who were raped suffered from PTSD symptoms and were thirteen times more likely to attempt suicide (Steinman, 2022). This statistic is likely higher in college students, as sexual violence occurs three times more frequently in college co-eds compared to women in general (RAINN, 2023).

The sharp rise in adolescent depression and suicidality is alarming, with data from 2021 indicating that almost three in five females report being persistently sad or hopeless for at least two weeks in the past year—a rate twice as high as males and the highest reported in a decade (CDC, 2023). Thirteen percent of adolescent females attempted suicide in the past year, a rate nearly double that of males (7%) but less than that for LGBTQ+ youth (22%; CDC, 2023). Suicide attempts are most likely to occur among students with previous abuse who are recently sexually assaulted (Keefe et al., 2018; Oh et al., 2021). An extreme form of sexual violence, rape, is associated with all mental health conditions and substance abuse (Blanco et al., 2022). In summary, mental health professionals should assess sexual abuse histories of college students presenting with affective symptoms, substance abuse, and/or suicidality.

The current politicized environment regarding SRH may exacerbate obstacles for college students to access resources that address physical trauma and emotional impact of a sexual assault. Mental health counseling and supportive care is critical to address resultant anxiety, PTSD, and depression associated with sexual violence. The emotional burden of restrictions in access to contraception, emergency contraception, and medication and surgical abortion heightens concern for the mental health and well-being of college students (Jerman et al., 2017). This distress is further exacerbated by the repeal of *Roe v Wade* in the *Dobbs v Jackson Women’s Health Organization* U.S. Supreme Court ruling and resultant cascading SRH restrictions in nearly half of the nation.

## College Student Sexual Health Promotion and Violence Prevention

The Campus Sexual Violence Elimination Act (Campus SaVe Act; Newlands & O’Donohue, 2016) was established in 2013 as an amendment to the Cleary Act to initiate greater transparency and require campus-wide sexual violence prevention and education programs. Despite this federal mandate, the dearth of evidence-based sexual violence prevention is an obstacle for effective program implementation on college campuses. College administrators are tasked with implementing programs that satisfy mandates while complying with Title IX. A review of university-provided sexual violence prevention programs revealed that they often take the form of trainings that occur during first-year student orientation (Amar et al., 2014). There is little support for their efficacy (Newlands & O’Donohue, 2016).

While reports indicate an increase in knowledge regarding the prevalence, impact, and consequences of sexual violence, this increase does not affect change in related behavior (Anderson & Whiston, 2005). In their review, Newlands and O’Donohue (2016) identify that the main approaches to sexual violence prevention are prevention programs with males; risk reduction programs with females; mixed-gender programs; and community level programs (e.g., bystander intervention programs). Prevention with males is arguably the most important approach to reduce rates of sexual violence given that males are the primary perpetrators (Newlands & O’Donohue, 2016), but this group is especially hard to reach (Rich et al., 2010). Prevention programs that incorporate a bystander framework where men see themselves as allies rather than potential direct perpetrators minimizes ‘backlash and animosity’ (Newlands & O’Donohue, 2016), attitudes that promote male resistance to attending rape prevention programs (Rich et al., 2010). Programs that address consent and communication show encouraging results as preventing sexual violence among college students (Newlands & O’Donohue, 2016; Salazar et al., 2014). Risk reduction programs involving females are better received and make fair contributions to sexual violence prevention; however, these programs overemphasize women’s risk behaviors and neglect teaching them how to recognize potential risk factors and behaviors of potential perpetrators (Newlands & O’Donohue, 2016; Rozee & Koss, 2001). The mixed-gender prevention programs evaluated were less effective than prevention programs with men and risk reduction with women (Newlands & O’Donohue, 2016).

### Athletic Programs and Sexual Violence Prevention

Colleges and universities with prominent intercollegiate athletic programs are particularly vulnerable to scenarios exemplative of sexual violence (Wiersma-Mosley & Jozkowski, 2019), including the prestigious National Collegiate Athletic Association (NCAA) Division I program. Division I college campuses report significantly higher rates of violence against women (i.e., stalking, domestic, and dating violence, rape) compared to Division II, III, and universities with no athletic programs (Wiersma-Mosley & Jozkowski, 2019). Mandatory sexual violence prevention programs (e.g., Title IX sexual violence prevention programs) have proven largely ineffective (Stafford, 2004), especially with policy emphasis in protecting against potential legal liabilities for their respective institutions rather than practical, contextually relevant guidance on communication, appropriate behaviors, and obtaining consent. Students are more likely to report incidents to peers as opposed to their coaches or other authority figures (Carey et al., 2018), suggesting that peer-administered sexual violence preventive interventions may have promise when they involve students, student athletes, coaches, and trainers as program participants.

Creative strategies have been employed to address sexual violence on college campuses, such as utilizing theater and the arts to educate students about risk factors and prevention of sexual violence with some notable success (Fleckman et al., 2022). Digital technology has also been explored as a method to reduce sexual violence such as digital gaming and interactive virtual experiences used as educational tools to raise awareness (Baldwin et al., 2021). Peer education programs, identification of effective student leadership, and providing awareness for substance and alcohol abuse have also shown promise (Spencer et al., 2022). Bystander intervention programs involving peer group facilitators have been effective (Kirk-Provencher et al., 2023). Peer norms are important drivers of attitudes and behaviors, underscoring the importance of confidentiality and privacy in sexual health service settings to avoid shame and stigma (Cassidy et al., 2018) and normalize and support sexual health service access. College student peer group settings may be ideal for interventions, such as athletic programs, sororities and fraternities, residential halls, and dormitories.

### Male Engagement

As noted previously, male college students are essential to engage in SRH promotion programs (Newlands & O’Donohue, 2016) yet they have low participation rates (Rich et al., 2010). More effort needs to be made to recruit and retain their active engagement. One approach is to increase content that they find highly relevant. This may include discussions of pornography, causes and prevention of testicular dysfunction, male infertility, and ED in adolescents and emerging adults. For example, male college students may be particularly interested in discussions about steroid derivatives that impact ED. Developed as a safer alternative to anabolic androgen steroids, an increasingly common cause of male SRH problems is recreational and athletic use of selective androgen receptive modulators (SARMs; Hoffman et al., 2009). Although the Food and Drug Administration in 2019 prohibited SARMs (also referred to as “prohormones”) from being manufactured and sold commercially, they are accessible through the Internet and unregulated (Efimenko et al., 2021; Machek et al., 2020). SARMs are popularly used because of their ease of oral administration and reputation as a novel recreational performance enhancing compound (Van Wagoner et al., 2017) that increases skeletal muscle size and function (Solomon et al., 2019). Its high abuse profile has led to widespread use in doses several times higher and longer duration than recommended (Machek et al., 2020), the suspected result of which could cause hypogonadal symptoms in men (e.g., lethargy, mood swings, ED, reduced libido, concentration problems, and compromised fertility; Efimenko et al., 2021; Guay & Davis, 2002) and risk for permanent masculinization among women (Machek et al., 2020), although more research is needed (Efimenko et al., 2021).

### Successful Preventive Intervention Components

According to the CDC’s report, *Sexual Violence on Campus: Strategies for Prevention* (Dills et al., 2016), comprehensive campus-based prevention strategies implemented on an individual level should focus on building healthy relationship skills, establishing positive norms about gender and sexuality, incorporating bystander intervention, and preventing violence through multiple informed, interactive sessions (Dills et al., 2016). There is limited research to support the effects of sexual consent programming in college-aged students, but it is more efficacious when taught within school-based sex education programs (Olson, 2022). Furthermore, effects over time are observed such that participation in sexual health programming before the age of 18 was found to reduce risk of sexual assault victimization in college (Santelli et al., 2018). A meta-analysis concluded that longer interventions are more effective than brief interventions in altering both rape attitudes and rape-related attitudes (Anderson & Whiston, 2005).

### STI and COVID-19 Prevention

As a result of the pandemic, health providers and researchers must redefine risk to include transmission of both STIs and other viruses (Bowling et al., 2022; Kassel, 2021), including COVID-19. Although not a STI, close contact with an infected person can result in COVID-19 transmission (Avert, 2021). Sexual health programs for college students should address COVID-19 to decrease risks (Evans, 2020). The mental health sequela of COVID-19 also needs to be incorporated into sexual health interventions, given evidence that emotional health influences sexual behaviors in this population (Firkey et al., 2022). Most college students consider COVID-19 to be a serious threat but do not report high perceived susceptibility to the virus for themselves (Maziarz & Askey, 2022), contributing to less protective behaviors.

### SRH Programs for Diverse Populations

Based on the extant research, creating and implementing comprehensive evidence-based sexual and mental health promotion programs that affect attitudinal and behavioral changes specifically within college students are warranted. Comprehensive sexual health prevention programs—particularly those that are non-heteronormative—should prioritize enrolling oppressed and/or minority college students given their disproportionate rates of STI and HIV infection. Interventions that are gender and sexuality focused as well as those that are racially/ethnically, culturally, and ability relevant administered at various points during the course of the college experience (e.g., booster sessions to help maintain intervention gains) could be promising in increasing sexual and mental health and reducing sexual violence on college campuses, especially while COVID-19 continues to have a substantial health impact. Like racial/ethnic minority students, the LGBTQ+ community may, among other challenges, often juggle multiple identities and oppression. This can discourage them from participating in sexual and mental health intervention programs if they feel excluded because of their background or sexual identity. Passive marketing approaches for program recruitment (e.g., distributing flyers or brochures along with other orientation materials, posting information online) may fail to reach the intended audiences, especially early in the academic year when new students may feel overwhelmed with information (Cassidy et al., 2018). Targeted culturally and contextually relevant outreach is important for SRH promotion programs to be inclusive and successful for diverse populations.

Although intervention research continues to focus heavily on European descent youth (Pina et al., 2019), colleges are ideally suited to provide equity in sexual and mental health education and access. The field of clinical psychology is progressing toward a person-centered approach that contextualizes psychological distress and well-being within an ecological approach; in order to target the impacts of oppressive systems for marginalized communities, liberation psychology may be an effective foundation (Singh & Gudiño, 2023). It decenters Eurocentric psychology, extends beyond coping and resiliency to emphasize the importance of healing from oppression, and promotes equity, empowerment, self-determination, community solidarity, and positive psychological health (Comas-Díaz et al., 2019; Watts & Flanagan, 2007). These are components that would be important in enhancing sexual health among college students from oppressed (e.g., racial/ethnic minorities, females, sexual minorities, people living with disabilities) populations who are well known to suffer disproportionately from sexual violence and compromised sexual and mental health outcomes (Basile et al., 2016; Stockman et al., 2015).

## Future Directions

College has been identified as the ideal setting for innovative, campus-wide programming to increase student access to resources that promote sexual and mental health and prevent unintended outcomes, like STIs (CDC, 2022a), HIV, unintended pregnancy, and sexual violence. The rates of sexually active college-aged people, sexual risk behaviors (e.g., casual sex; multiple, concurrent, or overlapping sex partners; sex under the influence of alcohol or drugs), sexual violence, and STI/HIV prevalence highlight the need for SRH education for college students (Wong et al., 2019). However, a review of colleges and universities showed that although the majority provided sexual assault prevention programs, only a small proportion (i.e., mostly public and large institutions) provided sexual health programs, concluding that there is an urgent need to invest more resources in comprehensive sex education (Shigeto & Scheier, 2022) that target unintended pregnancy, STI, HIV, and sexual violence prevention.

### Sexual and Reproductive Health Services

To facilitate health service access and utilization, SRH service providers and programs that prioritize being welcoming, confidential, and non-judgmental can help counter campus culture that promotes sexual risk behaviors (Cassidy et al., 2018). Youth from racial/ethnic minority groups, LGBTQ+ communities, and individuals with disabilities are more likely to report feeling less connected at school, although school connectedness is beneficial in reducing violence risk and should be promoted (CDC, 2023). Being more inclusive in program content may also help attract diverse participants, prepare students to engage with partners from varying backgrounds (e.g., racial-, ethnic-, sexual minorities, international students, students with disabilities), and enhance social and campus connectedness.

Sexual health curricula can capitalize on COVID-19 public health mandates already familiar to students (e.g., maintaining distance from others, using protection, getting tested regularly, immediately seeking treatment, inform recent contacts of potential exposure) and apply them to reducing risks for STIs, HIV, and other unwanted outcomes (Evans, 2020; Hauschild, 2021; Loeb et al., 2021). Technological advances made during COVID-19 helped many college students to become practiced in ordering COVID tests and checking test results on their smart phones or computers. Similar applications are being explored with STI/HIV testing on college campuses thereby addressing social barriers (e.g., embarrassment, shame, confidentiality, cost) attributed to poor rates of sexual health testing among emerging adults (Cuffe et al., 2016; Griner et al., 2022; Reeves et al., 2023). Traditional cognitive and behavioral risk prevention messaging should be expanded to include emotional aspects of sexual health (i.e., intimacy), particularly among college age females (Alexander et al., 2015). Including emotional components of SRH and risks may increase intervention effectiveness. Including developmentally targeted sexual health messaging that focuses on this population’s relational and intimacy experiences—or inexperience—is necessary to mitigate unwanted sexual health outcomes (Alexander et al., 2015).

Sexual and mental health providers can help students to create personalized definitions of safer sex behaviors that take their perceptions, prior experiences, familial/cultural backgrounds, and reducing virus transmission into account (Alexander et al., 2015). Reinforcing pride in ethnic identity, supportive social structure, empowerment, and strength-based approaches (which are proven to serve as protective factors against sexual risk behaviors) are suggested (Opara et al., 2020). Educational opportunities for parents on how to help reduce their student’s risk factors and preventive interventions for students (e.g., how to be in a healthy relationship) would be beneficial despite the advanced ages of their children. Positive outcomes have been found in those that promote healthy communication (e.g., sexual assertiveness), women empowerment, and self-awareness (Bhochhibhoya et al., 2021). Extending an intergenerational approach to comprehensive SRH prevention programming may include parents/caregivers, but also faculty and staff (e.g., athletic coaches, trainers) in ways that go beyond online sexual harassment prevention training mandated by colleges to prevent unwanted sexual and mental health outcomes.

Although parent–child sexual health communication may be awkward, positive parent–child communication and attitudes about sex and alcohol are protective factors against college students misusing alcohol and engaging in sexual risk behaviors (Coakley et al., 2017; Saint-Eloi Cadely et al., 2022). Parental guidance and support are protective factors against sexual violence (Hébert et al., 2019; Kaukinen, 2014). College students with a positive relationship with their parents/caregivers compared with those who have a harsh or negative relationship have fewer instances of unwanted sexual contact (Bhochhibhoya et al., 2021; Sutton & Simons, 2015). However, parents of ethnic minority youth are more reticent about speaking to their children about sex (Lantos et al., 2019) and may focus more on restrictive reproductive messages (Manago et al., 2014). Addressing the needs of minority college students who are highly vulnerable to infection is paramount if the prevalence of HIV/STIs in this population is to decrease significantly. One approach worth investigating is parent sexual health education. Given that parents/caregivers may not have had sex education since they were in high school perhaps decades ago, there may be a bidirectional effect if they participate in SRH education programming. It may be advantageous for the student because their parent/caregiver is better informed and may thus support their SRH, but it also may positively affect the older generation themselves in promoting their personal SRH (thus reducing risk for STIs/HIV, sexual violence). Future research should explore an intergenerational approach to SRH prevention programming among adolescents and emerging adults, involving parents and/or coaches, athletic trainers, and other collegiate staff.

## Implications for Clinical Psychologists

Clinical psychologists have historically been trained in a deficit or disease model of human functioning, focusing on the absence of illness and disability (Cooke et al., 2016; Seligman & Csikszentmihalyi, 2000) yet in contrast, positive psychology has emerged with a focus on psychological well-being and quality of life (Delle Fave et al., 2011; Gómez-López et al., 2019; Ryff & Keyes, 1995). Although there is variation found within the eudaimonic approaches to understanding well-being (Cooke et al., 2016), engaging in positive romantic relationships in adolescence and emerging adulthood is a key component, including interpersonal attachment, belongingness, relatedness, intensity, affection, and erotic sexual engagement (Gómez-López et al., 2019). In their review, Gómez-López et al. (2019) conclude that emerging adults who engage in healthy romantic relationships are happier, have more life satisfaction, display greater positive affect, have higher self-esteem, and experience less mental and physical illness than single people. Emerging adults attend college for education which should develop a life-long passion for learning and personal growth, including investing in one’s sexual, reproductive and mental health, and well-being throughout adulthood.

An approach that incorporates well-being and positive psychology may promote school campuses and clinical settings that move from shame and guilt to normalizing the spectrum of sexual activity—including inactivity and abstinence—to more fully engage adolescents and emerging adults. Preventive interventions should focus not only on preventing unwanted outcomes (i.e., pregnancy, STI, HIV, sexual violence, trauma) but also promoting desired results, like building social skills that form the basis of rewarding interpersonal and romantic relationships. It is important to consider that social skill development may have been delayed or impeded as an iatrogenic effect of the dependence on technology (rather than face-to-face interactions) that resulted from COVID-19 pandemic induced distance learning and social distancing. Clinicians and interventionists should address the potential need for enhanced communication skills, conflict resolution, assertiveness training, and practical, age-appropriate ways to obtain and maintain consent. Given the increased rates of anxiety, depression, and alcohol and substance use reported among adolescents and young adults during and after the onset of COVID-19, it is important for psychologists to assess and treat depression, anxiety (including social anxiety), trauma, and substance use among college-aged populations.

The recent U.S. Supreme Court decision that severely limited women’s reproductive health rights (*Dobbs v Jackson Women’s Health Organization*) is merely the most visible recent impetus for the growing need for psychologists to expand their definitions of health to include sexual health by taking a comprehensive sexual history along with a general history of mental health systems. The stress and anxiety among adolescents and young adults that the ongoing cascading changes in access to SRH options have created need to be better researched and treated. In a clinical setting, it may begin with an assessment of current stressors, anxiety, depression, substance use, and a sexual health history. That history might reveal sexual abuse and trauma experienced beginning in childhood and sometimes continuing into adulthood (Wyatt, 1992; Wyatt et al., 1992). Taking a history of sexual and intimate violence is increasingly being included in clinical psychology settings and training programs using measures like the Adverse Childhood Experiences Scale (ACES) and the UCLA Life Adversities Screener (LADS; Liu et al., 2015). Experiencing these types of violence have been associated with health and mental health problems that may increase risks of STIs, HIV, unintended reproductive outcomes, irritable bowel syndrome, anxiety, depression, and post-traumatic stress (Pemberton & Loeb, 2020).

Psychologists and other health providers need to be more attuned to the context of asking sensitive (i.e., sexual, reproductive, violence) questions that may facilitate responses to design effective treatment plans (Wyatt et al., 1992). The content and delivery (e.g., tone, body language, personal comfort) need to be comfortable, non-judgmental, without bias (especially regarding racial/ethnic and sexual minorities, people with disabilities, and diverse age groups), and culturally and contextually relevant. Clinicians may benefit from an enhanced awareness of their own SRH stereotypes, biases, comfort, and knowledge. Supervision and continuing education may help.

Psychology training programs may provide opportunities to observe interviews about consensual and nonconsensual sexual experiences included in sexual health assessments and integrate them into psychological assessments and clinical interventions. Supervisors and mentors may provide sexual and mental health education to trainees to prepare them for comprehensively treating and promoting mental health outcomes. The training should be factual; address the anatomy and physiology of sexual health for diverse individuals; explain how sexual illnesses are transmitted, detected, and diagnosed; discuss how to protect against unwanted sexual outcomes; include how to take sexual and violence histories and how to assess sexual and relationship satisfaction. Trainees and experienced clinicians should be cognizant of when and how to refer clients to other professionals (e.g., child protective services, law enforcement, college Title IX offices, gynecologists, urogynecologists, pelvic floor therapists, urologists, rape counselors, family planning clinics). Because SRH are essential components of overall well-being and quality of life for adolescents and emerging adults into later adulthood, it is important that renewed attention and efforts are made by psychologists and mental health providers that target their promotion in this COVID-19 era. Concentrated efforts to ameliorate the adverse effects are warranted and timely, and should not be relegated to clinicians with a subspecialty of sexual health but become more mainstream in clinical psychology.
